# Mucin 17 inhibits the progression of human gastric cancer by limiting inflammatory responses through a MYH9-p53-RhoA regulatory feedback loop

**DOI:** 10.1186/s13046-019-1279-8

**Published:** 2019-07-01

**Authors:** Bing Yang, Aiwen Wu, Yingqi Hu, Cuijian Tao, Ji Ming Wang, Youyong Lu, Rui Xing

**Affiliations:** 1 Laboratory of Molecular Oncology, Key Laboratory of Carcinogenesis and Translational Research (Ministry of Education), Peking University Cancer Hospital & Institute, Fu-Cheng Road 52#, Hai-Dian District, Beijing, 100142 China; 2 Cancer and Inflammation Program Center for Cancer Research, National Cancer Institute at Frederick, Frederick, MD USA

**Keywords:** Inflammation, Gastric cancer, MUC17, Survive

## Abstract

**Background:**

Mucins are key components of the mucosal barrier in the stomach that protects epithelia from carcinogenic effects of chronic inflammation. Analysis of The Cancer Genome Atlas database indicated that mucin-17 (MUC17) was more highly expressed in gastric cancer (GC) specimens, with favourable prognosis for patients. To explore the underlying mechanisms, we investigated the potential role of MUC17 in controlling chronic gastric inflammation.

**Methods:**

We initially quantified the expression of MUC17 and inflammatory factor, as well as the association of MUC17 with survive in GC using immunohistochemistry. To establish how the inflammatory factors affect MUC17 expression, we explored luciferase reporter, chromatin immunoprecipitation (ChIP), and electrophoretic mobility shift (EMSA) assays. The role and mechanism that MUC17 plays in inflammation-induced cell proliferation was examined in AGS cells with reduced *MUC17* expression and MKN45 cells overexpressing a truncated *MUC17*.

**Results:**

We found *MUC17* was induced by inflammatory cytokines in GC cells via CDX1upregulation. MUC17 thus inactivated NFκB to inhibit GC cell proliferation in response to pro-inflammatory cytokines. We also revealed that the function of MUC17 was dependent on its conserved epidermal growth factor domain and on downstream sequences to enable its interaction with myosin-9, resulting in a sustained regulatory feedback loop between myosin-9, p53, and RhoA, and then activation of p38 to negatively regulate the NFκB pathway in GC cells. This mechanism was also confirmed in vivo.

**Conclusions:**

Our study demonstrates MUC17 as a GC suppressor protein which has the therapeutic potential for human GC.

**Electronic supplementary material:**

The online version of this article (10.1186/s13046-019-1279-8) contains supplementary material, which is available to authorized users.

## Backgound

Gastric cancer (GC) is one of the most common and aggressive cancer types throughout the world [1]. Most GC associates with dysfunction of the mucosal barrier in the stomach, the first line of defence against pathogens and inflammation. Impairment of the gastric mucosal barrier often leads to chronic inflammation, a major contributor to the development of GC [2]. One of the main components of the mucosal barrier that protects the underlying stomach epithelium is the mucin family of high molecular-weight glycosylated proteins [3, 4], with 21 mucin genes identified (*MUC1*-*MUC21*) so far. In recent years, mucins have also been found to be both therapeutic targets and biomarkers predicting the prognosis of various cancers [5]. Studies suggest that mucins may act as tumour-suppressing genes. For example, *MUC2*^−/−^ mice develop colon adenomas, suggesting that MUC2 is involved in the suppression of colorectal cancer [6]. Further, their expression is regulated by inflammatory stimulants. For example, IL1β and IL17A both promote MUC5B expression in target cells [7]. IL8 (CXCL8) has also been shown to regulate MUC5AC expression [8], while IL4 and IL13 both increase *MUC2* mRNA expression [9]. However, the biological role of mucins in GC development, and association with inflammation is not well understood.

To establish the link between mucins and inflammation in GC progression, we analysed transcriptional data retrieved from The Cancer Genome Atlas (TCGA) and discovered that *MUC17* was consistently expressed at high levels in GC tissues, which is associated with favourable patient survival after surgery. We therefore investigated the expression of *MUC17* in a patient cohort to more precisely establish a link between MUC17 and inflammation in the development of GC. Here, we report that MUC17 inhibits inflammatory response in the stomach to protect the epithelial cell from carcinogenesis.

## Materials and methods

### Clinical samples

Experiments were conducted in accordance with the Helsinki Declaration 2013 and were approved by the Institutional Ethical Standards Committee of Beijing Cancer Hospital. Written informed consent was obtained from all patients. All GC samples were collected from the Beijing Cancer Hospital and were evaluated by a centralized pathological review group.

### Cell lines and transfection

AGS cells were purchased from the American Type Culture Collection (ATCC, Manassas, VA, USA), MKN45 cells were purchased from the Cobioer (Nanjing, China). Mycoplasma testing was negative. AGS and MKN45 cells were cultured in Dulbecco’s modified Eagle’s medium (Gibco, Grand Island, NY, USA) with 10% or 20% fetal bovine serum (Gibco). Cells were maintained at 37 °C under 5% CO_2_.

The transfection was performed by using Lipofectamine 2000 (Invitrogen, Carlsbad, CA, USA) following the manufacturer’s instructions.

### Immunohistochemistry

GC specimen without chemotherapy and radiotherapy before surgery were random collected. Immunohistochemistry was performed using a Dako Real Envision Detection System (Dako, Glostrup, Denmark). Slides were incubated with a primary antibody specific to MUC17 (Sigma-Aldrich, St. Louis, MO, USA), followed by incubation with a biotinylated secondary antibody and enzyme conjugate (Dako). These then stained with 3,3′-diaminobenzidine and counterstained with hematoxylin. The sample size was determined using the positive MUC17 staining rates in a small preliminary experiment using PASS (NCSS, East Kaysville, UT, USA). A pathologist blinded to the purpose of the study read the microarray in this study and gave an unbiased report. Our study was conducted in accordance with the Helsinki Declaration, and was approved by the Institutional Ethical Standards Committee with informed consent from patients.

### RNA isolation and polymerase chain reaction (PCR)

Total cellular RNA was isolated from cells using TRIzol Reagent (Invitrogen) and reverse transcribed using the cDNA reverse transcription kit (Invitrogen). The expression levels of *MUC17* and *CDX1* mRNA were analysed using SYBR Green qPCR reagent (Transgen Ltd., Beijing, China) using an ABI7500 qPCR System (Applied Biosystems, Foster City, CA, USA). The primer sequences are listed in Additional file 3: Table S1. The relative expression levels of each gene were normalized against an actin endogenous control using the 2^-ΔΔ^Ct method.

### Luciferase assay

Luciferase reporter assays were performed using a Dual-Luciferase Reporter Assay System (Promega, Madison, WI, USA) with the pGL3 basic luciferase reporter system. The promoter sequence was chosen by using UCSC database based on the peaks of H3K4Me3 and H3K27AC. The promoter regions were cloned into pGL3-basic plasmid using SmaI and XhoI (Endonucleases were purchased from NEB, MA, USA; T4 DNA ligase was purchased from Promega). Promoter-specific luciferase constructs (the primers used for construction of the plasmid are listed in Additional file 3: Table S1) and empty pGL3-basic were co-transfected with a control Renilla luciferase construct into cells using Lipofectamine 2000 (Invitrogen). The luciferase signal was first normalized to the Renilla luciferase signal and then normalized to the signal from the empty pGL3 plasmid. All experiments were performed independently at least three times.

### Electrophoretic mobility shift assay (EMSA)

Nuclear extracts were first isolated from AGS cells and then nuclear proteins (3 μg) were mixed with biotin-labeled probes (Additional file 3: Table S1) containing the *MUC17* consensus sequence (50fmol). These were then incubated at 25 °C for 20 min. The protein-DNA mixtures were separated from unbound probe using a 6% polyacrylamide gel at 4 °C for 2 h in a Tris-glycine-EDTA running buffer. The gel was then transferred and detected using an enhanced chemiluminescence (ECL) detection system (Sage Creation, Beijing, China).

### Chromatin immunoprecipitation (ChIP) assay

ChIP assays were performed using an Agarose CHIP Kit (ThermoFishier, Carlsbad, CA, USA) and the manufacturer’s protocols. The primary antibodies used were specific to CDX1 (Abcam, Cambridge, MA, USA) and Histone H4 (acetyl K5) (Abcam).

### Western immunoblotting

SDS-PAGE and western blots were performed using standard protocols. Antibody binding to bands was detected using an ECL detection system (Sage Creation). The primary antibodies used were specific to MUC17 (Sigma-Aldrich, St Louis, MO, USA), IL-1β (Bioworld, St Louis Park, MN, USA), IL-8 (Bioworld), cdx1 (Abcam), pJNK (Affinity biosciences, Cincinnati, OH, USA), pERK (Santa Cruz, Dallas, TX, USA), p38 (Cell Signaling Technology, Danvers, MA, USA), pp38 (Cell Signaling Technology), PPARγ (Affinity biosciences), pp65 (ZSGB-Bio, Beijing, China), p65 (ZSGB-Bio), p21^waf^ (Proteintech, Rosemont, IL, USA), p53 (ZSGB-Bio), Myh9 (Abcam, Cambridge, UK), and Actin (Sigma-Aldrich).

### RhoA activity

The activation of RhoA pathway was detected by Active Rho Pull-down and Detection Kit (ThermoFishier).

### Immunofluorescence

Cells were seeded on coverslips and fixed with 4% paraformaldehyde. They were then permeabilized with 0.5% Triton X-100, blocked using 5% bovine serum albumin, and stained overnight with monoclonal antibody specific to p65 (ZSGB-Bio, Beijing, China) at 4 °C. After incubation with FITC-conjugated goat anti-rabbit IgG (ZSGB-Bio), cells were stained by 4′,6-diamidino-2-phenylindole (DAPI). Images were captured by fluorescent microscopy (LSM510; Zeiss, Toronto, ON, Canada).

### MTT and Colony formation assays

Cells were seeded onto 96-well culture plates and MTT (5 μg/mL) was added to the cells each day. MTT was removed after 4 h of incubation and then dimethyl sulphoxide (DMSO) added to solubilize the formazan product. Absorbency at 490/570 nm was assayed using a microplate reader (Biorad 680 ELISA; Biorad, Hercules, CA, USA). For colony formation, 500 cells were seeded on to 60 mm Petri dishes. After 2 weeks, cells were fixed using 4% paraformaldehyde for 10 min and stained by crystal viola for 20 min, and the colonies were numbered.

### BrdU incorporation assay

BrdU incorporation assay was performed using the BrdU Cell Proliferation Assay Kit (Cell signalling technology, MA, USA) and the manufacturer’s protocols.

### Cell cycle assays

Cells were fixed in 75% alcohol overnight at 4 °C and stained in a solution containing propidium iodide for 15 min, following the protocols of a cell cycle detection kit (Beyotime Ltd., Jiangsu Province, China). The cells were then analysed by flow cytometry (ArrayTM Bioanalyzer, BD Biosciences, San Jose, CA, USA).

### Tumourigenicity assays

Animals were randomized and the experimenter was aware that the animals were injected into two kinds of cells but was blinded to the outcome of each group. AGS cells (5 × 10^6^ cells suspended in 0.1 mL PBS) transfected with either shRNA against MUC17 or empty vector were injected subcutaneously into the axillary of five 4-week-old female Nod Scid mice. After 3 months, the tumors were locally injected with p38 inhibitor or DMSO. Animal experiments were performed in according with the National Institutes of Health Guide for the Care and Use of Laboratory Animals with protocols approved by the Animal Care and Use Committee at Peking University Cancer Hospital & Institute.

### Statistical analysis

The t-test or one-way analysis of variance (ANOVA) was applied to determine significant differences between groups. Two-way ANOVA was applied to determine significant differences between different treatments. For analysis of the immunohistochemical images, significant differences were determined with χ^2^ tests and SPSS 13.0 (IBM, Armonk, NY, USA). Any effects on survival were analysed using the Kaplan-Meier method. All comparisons were two-tailed and *P* values < 0.05 were considered significant.

## Results

### High *MUC17* mRNA expression is associated with better prognosis of GC

**Fig. 1:**
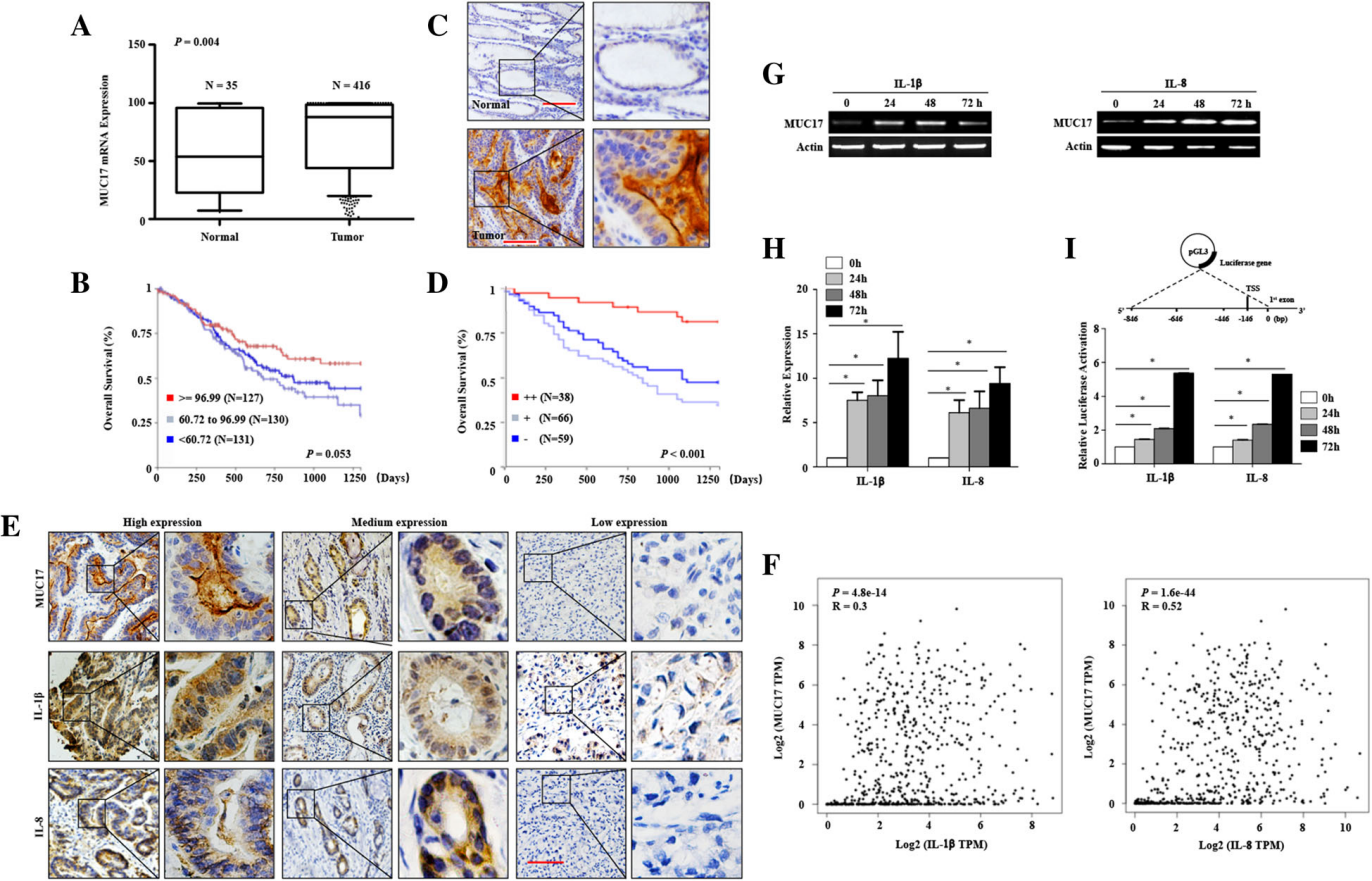
High expression of MUC17 is associated with improved prognosis in GC patient and inflammatory cytokines induce the expression of MUC17 in GC cells. **a** Higher *MUC17* expression was detected in GC tissues compared to normal tissues using information from the TCGA_GC database. The median was shown. **b** Higher levels of *MUC17* expression associated with improved prognosis in patients using information from the TCGA_GC database. **c** The expression of MUC17 in GC tissues and adjacent normal tissues detected by immunohistochemistry. Scale bar, 50 μM. **d** High levels of *MUC17* expression associated with favourable prognoses in our study cohort. **e** Co-expression of MUC17 with IL1β and IL8 in GC tissues detected by immunohistochemistry. Scale bar, 50 μM. **f** Analysis the expression association between MUC17 and IL1β (left) and IL8 (right) by using TCGA_GC transcriptional database. **g** Treatment with IL1β (left) and IL8 (right) increased the expression of *MUC17* determined by RT-PCR. **h** Treatment with IL1β and IL8 increased the expression of *MUC17* determined by qPCR. Error bars represent ± SD of three experiments (*, *P* < 0.05). **i** Upper, scheme of MUC17 promoter-luciferase fusion plasmids. Lower, treatment with IL1β and IL8 increased the activity of the *MUC17* promoter measured by luciferase assays. Error bars represent ± SD of three experiments (*, *P* < 0.05)

Our analysis of TCGA_GC database revealed that the mRNA levels of *MUC1* (*P* = 0.019), *MUC12* (*P* < 0.001), *MUC13* (*P* < 0.001), *MUC16* (*P* < 0.001), and *MUC17* (*P* = 0.004) were more highly expressed in GC tissues compared to normal tissues (Fig. 1a and Additional file 1: Figure S1). Kaplan-Meier survival analysis indicated that *MUC17* was the only mucin gene whose high expression was associated with better overall survival of GC patients (Fig. 1b). To validate the mRNA data, we assessed MUC17 protein in 163 GC tissue samples using immunohistochemistry (IHC). The clinical characteristics of the patients were listed in Additional file 3: Table S2. Based on the intensities IHC, we categorized the levels of MUC17 protein into high, medium, and low groups. The expression of MUC17 protein was consistently higher in GC tissues compared to adjacent normal tissue (Fig. 1c, Additional file 3: Table S3, *P* < 0.001). Kaplan-Meier survival analysis revealed that high expression of MUC17 was associated with better overall survival of patients in this cohort (Fig. 1d), which was confirmed by univariate analysis (*P* = 0.010, Additional file 3: Table S4). Multivariate Cox Proportional Hazard Analysis demonstrated that high expression of MUC17 protein was an independent prognostic predictor for GC (*P* = 0.016, Additional file 3: Table S4).

### Inflammatory mediators upregulate the expression of *MUC17*

One of the main functions of mucins is to protect the mucosal epithelial layer from direct exposure to substances including inflammatory mediators. We hypothesized that mucin expression in epithelial cells may be increased in response to inflammatory stimulation. We thus assessed the expression of various inflammatory mediators in GC tissues using IHC, and found that IL1β and IL8 (*P* < 0.001, *R* = 0.733; *P* < 0.001, *R* = 0.646), but not IL6 (*P* = 0.073, *R* = 0.433) levels associated with the expression of MUC17 protein (Fig. 1e). To confirm this correlation, we performed correlation analysis between IL1β/IL8 and MUC1,12,13,16 and 17 by using TCGA GC database. As shown in Fig. 1f and Additional file 2: Figure S2, compared with MUC1,12,13,16, MUC17 had more significant correlation with IL1β (*R* = 0.30, *P* < 0.001) and IL8 (*R* = 0.52, *P* < 0.001). To investigate a causal link between the expression of inflammatory factors and MUC17 in GC, we treated AGS cell line in vitro with IL1β and IL8. *MUC17* mRNA levels were increased 24 h after exposure, suggesting that these inflammatory cytokines upregulated *MUC17* mRNA expression (Fig. 1g and h). Luciferase activity assay using *MUC17* promoter indicated that IL1β and IL8 enhanced *MUC17* promoter activity in cells transfected with a reporter gene (Fig. 1i).

### CDX1 as a transcriptional factor that controls *MUC17* expression

**Fig. 2:**
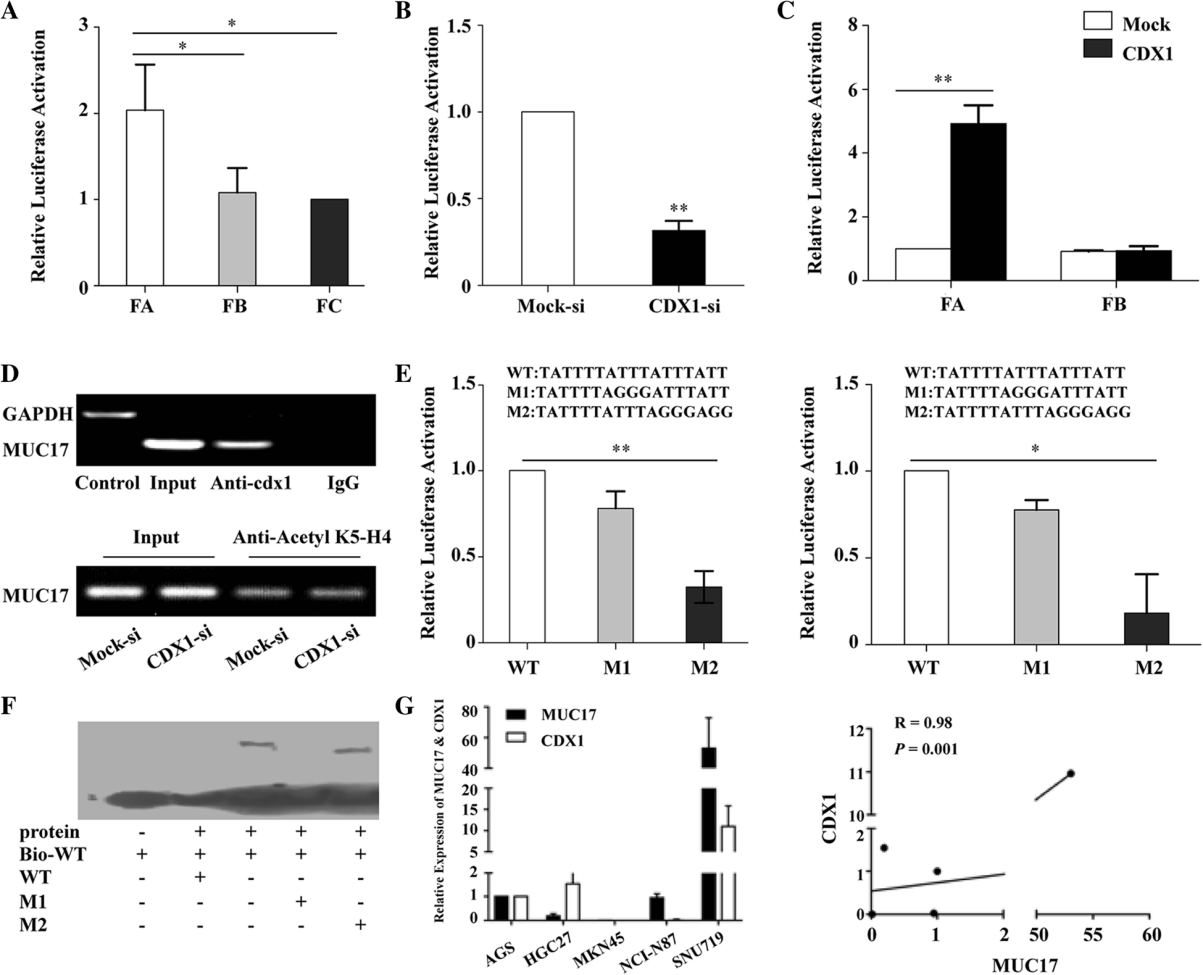
Cdx1 serves as a transcriptional factor of MUC17. **a** Luciferase assays using AGS cells transfected with one of three MUC17 promoter fragments with differently sized deletions (FA-FC). **b** The luciferase activity of the FA fragment in AGS cells transfected with a shRNA targeting *CDX1*. **c** The luciferase activity of FA and FB in MKN45 cells transfected with *CDX1*. **d** Upper, ChIP assay revealed that interacted CDX1 with the promoter region of *MUC17*. Lower, ChIP assay indicated knocked-down of CDX1 did not impact the transcriptional state of chromatin by using the antibody against H4 acetylated K5. **e** Luciferase assay using the FA fragment and its mutant derivatives transfected into AGS cells (left) and MKN45 cells overexpressing CDX1 (right). **f** EMSA assay confirmed that CDX1 binding to the second motif of the *MUC17* promoter. **g** The expression of *MUC17* and *CDX1* mRNA detected by qPCR in GC cell lines (left). The correlation between *CDX1* and *MUC17* expression in GC cell lines (right). Error bars represent ± SD of three experiments (*, *P* < 0.05; **, *P* < 0.001)

To further investigate the underling mechanisms, we transfected AGS cells with sequences of various lengths selected from the 5′ flanking region of *MUC17* gene attached to a luciferase reporter. These sequences included progressive deletions from 0.7 kbp upstream of the transcriptional start site, termed FA (− 846 bp), FB (− 646 bp), and FC (− 446 bp) (Fig. 1i). After transfection into AGS cells, there was a reduction in luciferase activity in FB compared to FA (*P* = 0.050, Fig. 2a), suggesting the location of a transcriptional promoter in this region (− 846 to -646 bp). Subsequent bioinformatics analysis indicated the presence of two CDX1-binding sites in the region.

To confirm the capacity of CDX1 to regulate *MUC17* expression in GC cells, we knocked-down *CDX1* in AGS cells using shRNA. There was a decrease in the luciferase activity in the *MUC17* promoter reporter, indicating that CDX1 directly affected the activity of the *MUC17* promoter (*P* < 0.001, Fig. 2b). We also overexpressed *CDX1* in MKN45 GC cells that expressing lower levels of CDX1, resulting in the enhancement of the luciferase activity of the FA region, but not the FB region (Fig. 2c). These results indicate that CDX1 promotes the expression of *MUC17* by binding to the promoter between − 846 and -646 bp in the 5′ region. To ascertain that *MUC17* was a downstream target of CDX1, an antibody specific to CDX1 pulled down the *MUC17* promoter region with CDX1 in ChIP assay (Fig. 2d **upper**). To exclude the possibility that knockdown of CDX1 impacts the transcriptional state of chromatin, we performed the ChIP experiment in sh-CDX1 conditions by using the antibody against H4 acetylated K5. As shown in Fig. 2d **lower**, the signal of MUC17 promoter was not significantly changed between Mock-si cells and CDX1-si cells, indicating knockdown of cdx1 did not impact the transcriptional state of chromatin.

To verify the CDX1 binding region in the *MUC17* promoter, two reporters fused *MUC17* promoter variants were constructed that mutated CDX1 DNA binding motif (5′-A, A/T, T, A/T, A, T). The luciferase activity of the M2 variant in AGS cells was reduced compared to wild-type (WT) and the M1 variant (Fig. 2e, **left**). In MKN45 cells that overexpressing CDX1, the luciferase activity of the M2 variant was also reduced compared to WT and the M1 variant (Fig. 2e**, right**). EMSA assay in which detected signal in the cells with M2 variant, but not in the WT nor M1 variant (Fig. 2f). These data demonstrate CDX1 as a transcriptional factor for MUC17 and binds to the second 5′-ATTTAT motif of its promoter.

We next evaluated the expression of *MUC17* and *CDX1* in GC cell lines using qPCR (Fig. 2g). The levels of *CDX1* expression were strongly correlated with *MUC17* expression (*R* = 0.980, *P* = 0.001). MEM database indicated co-expression of CDX1 and MUC17 in GC cells (*P* < 0.001).

**Fig. 3:**
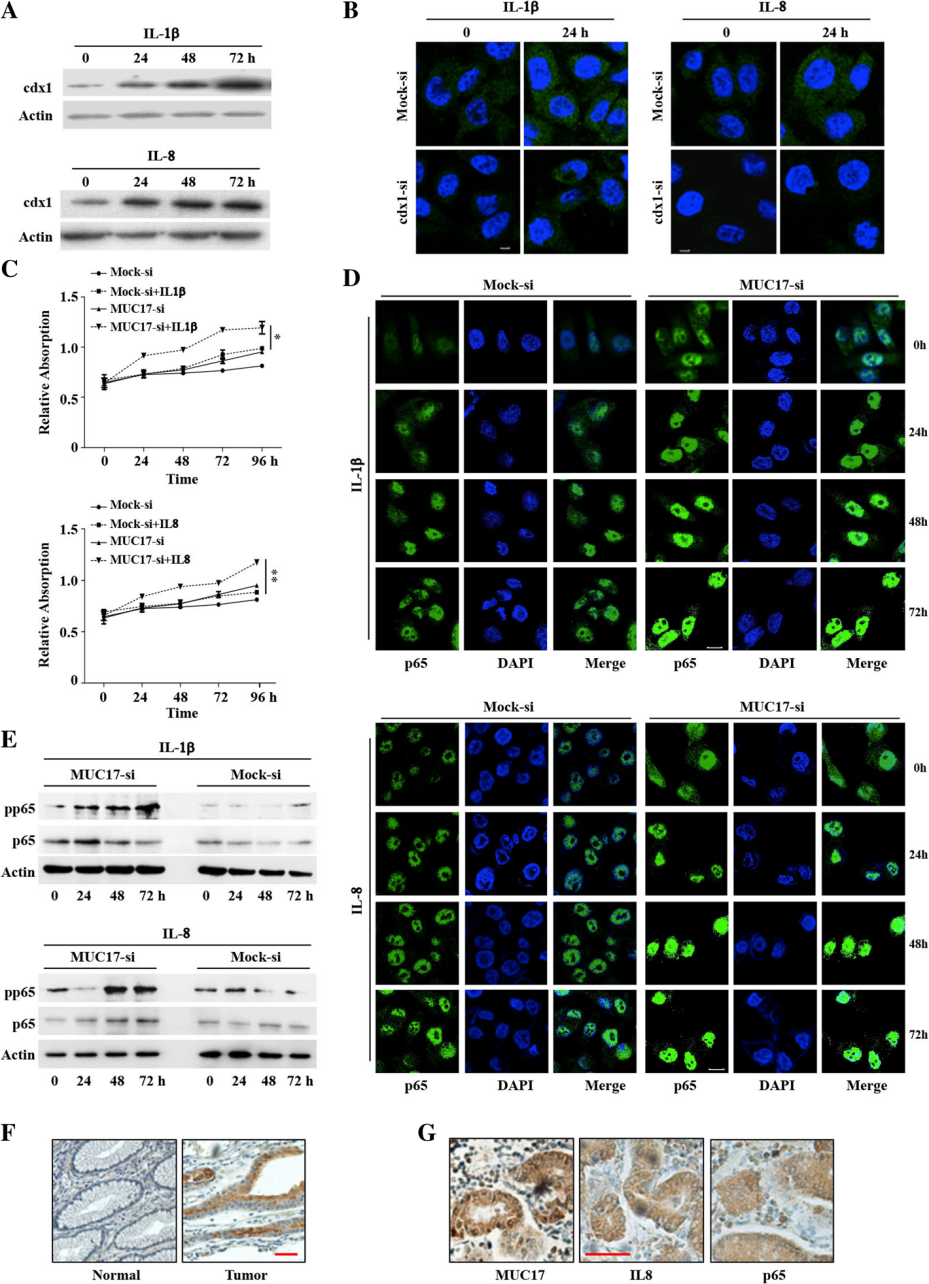
MUC17 protects GC cells against the effects of inflammatory cytokines. **a** Increased CDX1 expression induced by IL1β and IL8 in AGS cells, as determined by western blotting. **b** Immunofluorescence showed the failure of IL1β and IL8 to increase MUC17 expression in AGS cells transfected with a shRNA targeting *CDX1*. Scale bars, 10 μM. **c** Significantly increased MUC17-si cell growth rates induced by IL1β and IL8 compared to Mock-si cells in an MTT assay. Error bars represent ± SD (*P < 0.05, ** *P* < 0.001). **d** Increased p65 translocation to the nucleus in MUC17-si cells induced by IL1β (upper) or IL8 (lower) compared to Mock-si cells, detected by immunofluorescence. Scale bars, 10 μM. **e** Upregulating pp65 expression in MUC17-si cells induced by IL1β (left) or IL8 (right) compared to Mock-si cells, detected by Western blotting. **f** The expression of MUC17 in early stage GC tissues (right) and adjacent normal tissues (left). Scale bar, 50 μM. **g** In MUC17 highly expressed GC tissues, IL8 expression was negatively correlated with p65 expressed in nuclear. Scale bar, 50 μM

To investigate the role of CDX1 in inflammation-induced expression of MUC17, we examined the effect of inflammatory mediator on AGS cells. As shown in Fig. 3a, treatment with IL1β and IL8 increased the expression of CDX1. However, when CDX1 was knocked-down in AGS cells, IL1β or IL8 failed to increase the expression of MUC17 (Fig. 3b).

### MUC17 as biomarker of early stage GC protects gastric mucosa against the effects of inflammation

In AGS cells, with MUC17 knocked down (MUC17-si), IL1β and IL8 promoted more rapid growth of MUC17-si cells as compared to Mock-si cells (Fig. 3c). Since IL1β and IL8 promote cell growth through the NFκB pathway, we next measured the activation of this pathway connected to MUC17. Immunofluorescence assays showed that the level of the nuclear located NFκB p65 subunit (marker of NFκB activation) were lower in Mock-si cells than that in MUC17-si cells (Fig. 3d). After treatment with inflammatory cytokines, the levels of nuclear p65 remained higher in MUC17-si cells compared to that in Mock-si cells. To confirm the result of immunofluorescence, western blotting was employed. As shown in Fig. 3e, the signal of the phosphorylated p65 (pp65, marker of NFκB activation) were higher in MUC17-si cells than that in Mock-si cells. After treatment with inflammatory cytokines, the signal of pp65 remained higher in MUC17-si cells compared to Mock-si cells since 48 h. These results suggest that MUC17 protects against inflammatory cytokines induced epithelial cell proliferation by inhibiting the NFκB pathway.

Since inflammation is an early event of GC, we evaluated whether MUC17 may as a biomarker of early stage GC. IHC in 42 early stage GC tissues and adjacent normal tissues demonstrated that the expression of MUC17 was consistently higher in early stage GC tissues compared to adjacent normal tissue (Fig. 3f, Additional file 3: Table S5, *P* < 0.001, the clinical characteristics of the patients were listed in Additional file 3: Table S6). To evaluate the effect of MUC17 on limiting IL8 response though inhibiting NFκB pathway, we detected the expression of IL8 and p65 in the early stage GC tissues in which also detected the expression of MUC17. As shown in Fig. 3g, in MUC17 highly expressed GC tissues, IL8 expression was negatively correlated with p65 expressed in nuclear (*R* = − 0.319, *P* = 0.040).

### MUC17 induces GC cell cycle arrest

**Fig. 4:**
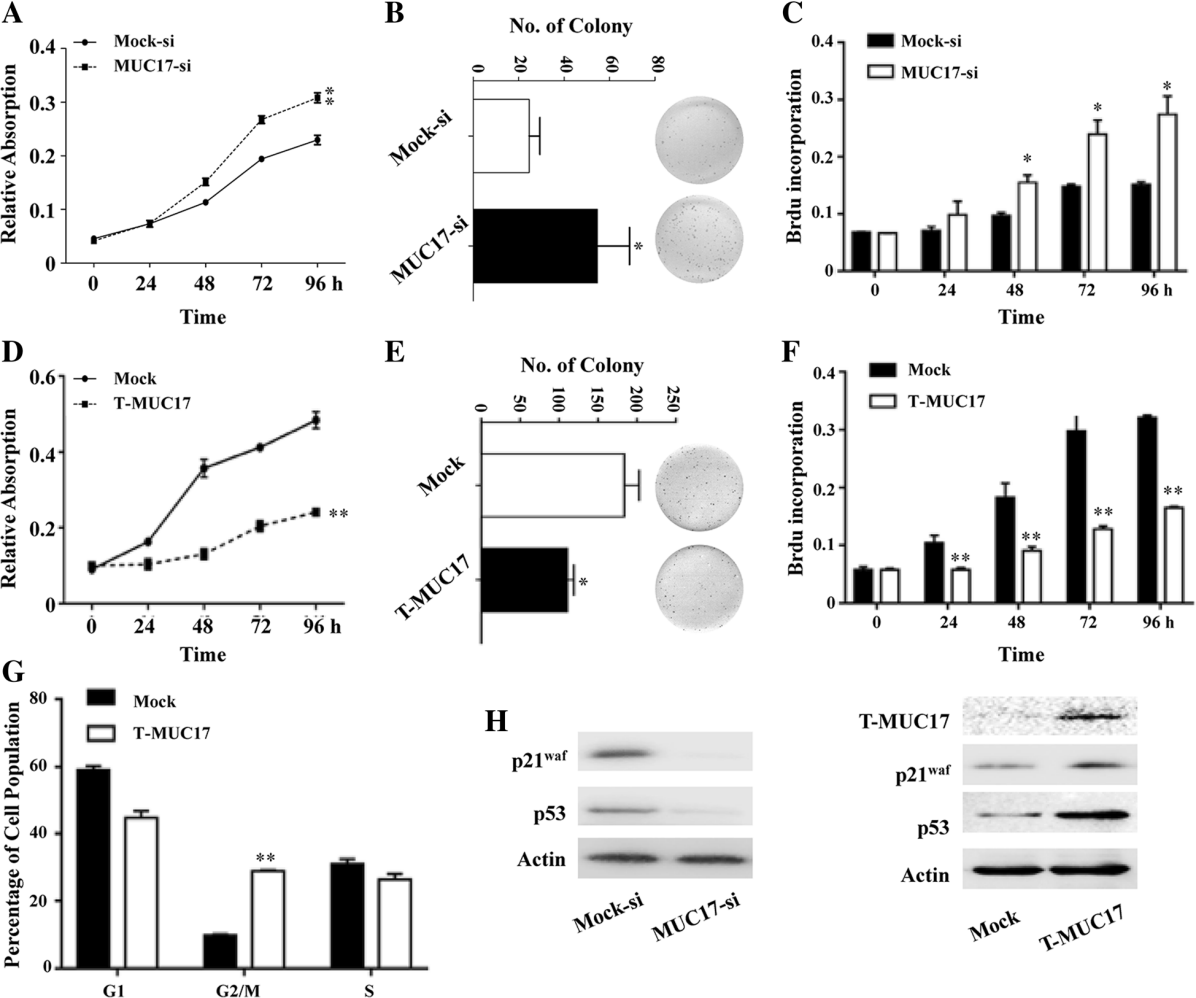
MUC17 inhibits GC cell proliferation through its EGF domain and following sequence. **a** Knock-down of *MUC17* promoted GC cell proliferation in AGS cells measured by MTT assay. **b** Knock-down of *MUC17* increased AGS cells proliferation measured by colony formation assay. **c** Knock-down of *MUC17* promoted BrdU incorporation in AGS cells measured by BrdU incorporation assay. **d** Overexpression of truncated MUC17 inhibited MKN45 cells proliferation measured by MTT assay. **e** Overexpression of truncated MUC17 inhibited MKN45 cells proliferation measured by colony formation assay. **f** Overexpression of truncated *MUC17* inhibited BrdU incorporation in MKN45 cells measured by BrdU incorporation assay. **g** Overexpression of T-MUC17 induced G2/M phase cell cycle arrest of MKN45 cells. **h** Knock-down of *MUC17* decreased the expression of G2/M phase arrest marker (left) and overexpression of truncated *MUC17* increased the expression of the markers (right). Error bars represent ± SD of three experiments (*, *P* < 0.05; **, *P* < 0.001)

To clarify the mechanism by which MUC17 inhibits the NFκB pathway in GC cells, we investigated the functions of MUC17 on GC cell growth. MTT and colony formation assays showed that MUC17-si cells grew faster than Mock-si cells (Fig. 4a and b). To further confirm this phenomenon, the BrdU incorporation assay was performed. As shown in Fig. 4c, knocked down of MUC17 significantly increased the incorporation of BrdU since 48 h. Structurally, MUC17 has a signal peptide, followed by a 59-amino acid tandem repeat, two EGF-like domains, an SEA domain, a hydro- phobic transmembrane domain, and an 80-amino acid C-terminal cytoplasmic domain that contains potential serine and tyrosine phosphorylation sites. According to the TCGA_GC database, while MUC17 is highly mutated in GC, the gene encoding EGF and its downstream region are conserved, suggesting that these domains may be critical for MUC17-mediated inhibition of cell proliferation. To test this hypothesis, we constructed a truncated variant of *MUC17* (that included the signal peptide, EGF domain, and downstream sequence) and overexpressed this variant in MKN45 cells (T-MUC17). T-MUC17 cells grew more slowly than Mock cells (Fig. 4d and e). Consistently, overexpressed T-MUC17 significantly decreased the incorporation of BrdU since 24 h (Fig. 4f). Flow cytometry showed that T-MUC17 induced cell G2/M phase arrest (Fig. 4g). To confirm that MUC17 promoted G2/M phase arrest, several molecular markers were examined, including p53 and the downstream p21^waf^ protein. p53 and p21^waf^ levels were reduced in MUC17-si cells as compared to Mock-si cells. In contract, p53 and p21^waf^ levels were higher in T-MUC17 cells as compared to Mock cells (Fig. 4h). These data indicated that MUC17 inhibits GC cell growth by inducing G2/M phase arrest, likely involving conserved EGF domain and the downstream sequence.

### MUC17 inhibits inflammation in GC cells by activating the p38 pathway

**Fig. 5:**
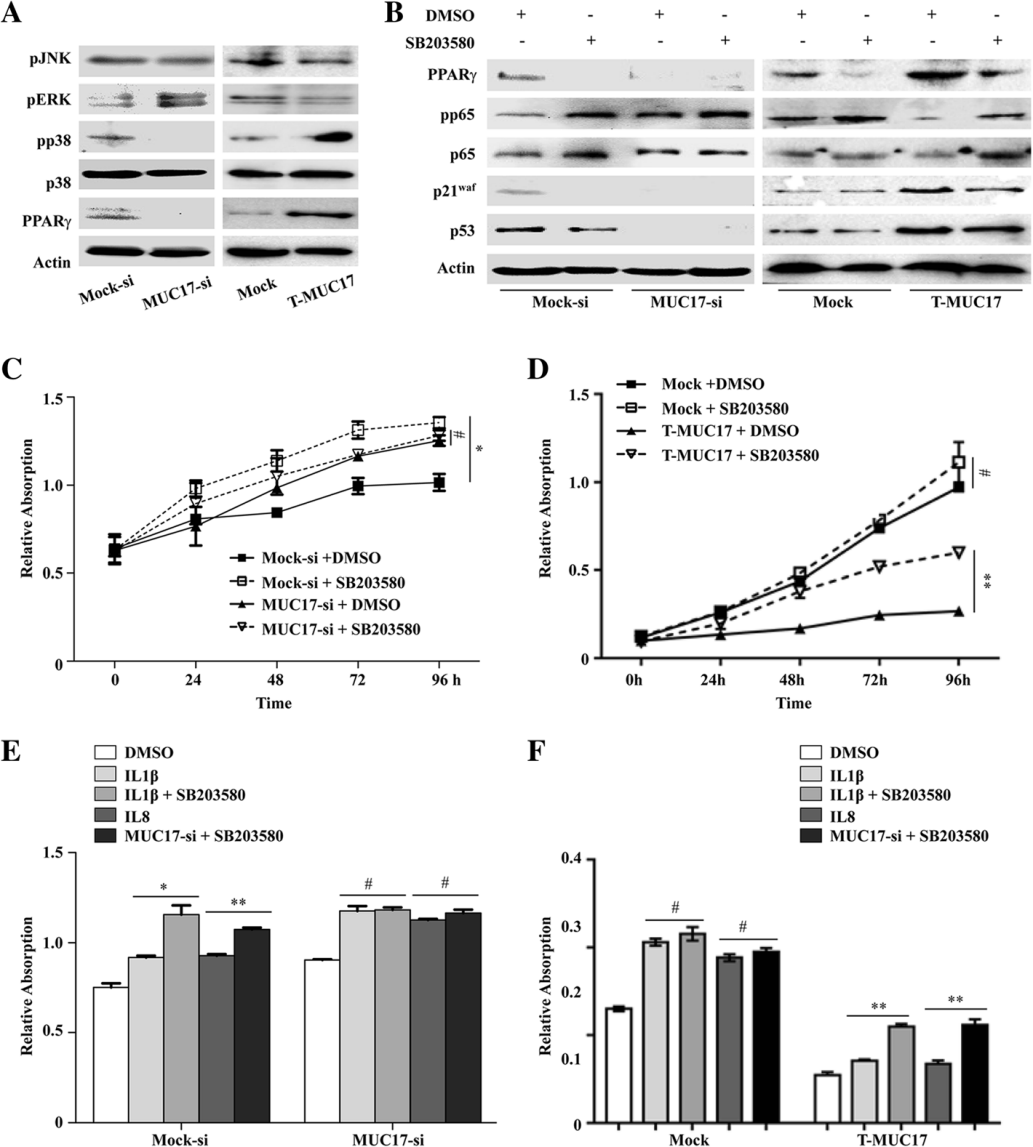
MUC17 protects GC cells against inflammatory stimulation by regulating the p38 pathway. **a** MUC17 regulated the expression of pJNK, pERK, and pp38 MAPK in *MUC17* knocked-down AGS cells (left) and truncated MUC17 overexpressed MKN45 cells (right). **b** The effect of the p38 inhibitor SB203580 on the expression of NFκB pathway proteins and G2/M phase arrest markers in *MUC17* knocked-down AGS cells (left) and truncated MUC17 overexpressed MKN45 cells (right). **c** SB203580 decreased the effects of MUC17 on inhibiting AGS cells proliferation. **d** SB203580 decreased the effects of truncated MUC17 on inhibiting MKN45 cells proliferation. **e** SB203580 decreased the protect effects of MUC17 on AGS cells under inflammatory stimulation. **f** SB203580 decreased the protect effects of truncated MUC17 on MKN45 cells under inflammatory stimulation. Error bars represent ± SD (#*P* > 0.05, *P < 0.05; **P < 0.001)

To explore the mechanism by which MUC17 protects against the effects of inflammation and induces G2/M arrest, the phosphorylated expression levels of three major mitogen-activated protein kinases (MAPKs), c-JUN N-terminal kinase (JNK), extracellular signal-regulated kinase (ERK), and p38, were measured, due to the EGF like domain MUC17 has. Knock-down of *MUC17* in GC cells slightly increased pERK, decreased pp38, and had no effect on pJNK (Fig. 5a, **left**). GC cells with *MUC17* knock-down also expressed lower levels of PPARγ, the downstream target of p38. Truncated *MUC17* transfected MKN45 cells (T-MUC17) were used to validate the role of the EGF-like domain and downstream sequence in the activation of MAPKs. T-MUC17 increased pp38 and PPARγ expression, but had no effect on pERK and pJNK expression (Fig. 5a, **right**). These data suggest that MUC17 activates p38 pathway through conserved EGF-like domain and downstream sequence.

To further investigate whether MUC17 protects GC cells against inflammation and induces G2/M cell arrest through the p38 pathway, we used a p38 inhibitor (SB203580). In Mock-si cells, SB203580 treatment decreased PPARγ and p21^waf^ expression but increased p65 and pp65 expression with no effect on p53 expression (Fig. 5b, **left**). SB203580 had no effect on the expression of these proteins in MUC17-si cells. In T-MUC17 cells, SB203580 treatment decreased PPARγ and p21^waf^ expression and increased the expression of p65 and pp65 without effect on p53 expression. SB203580 did not affect the expression of these proteins in Mock cells (Fig. 5b, **right**). Consistent with these changes, SB203580 promoted the proliferation of Mock-si cells but not MUC17-si cells (Fig. 5c). SB203580 also promoted the proliferation of T-MUC17 cells but not Mock cells (Fig. 5d). We then co-treated the cells with SB203580 and either IL1β or IL8. SB203580 increased the effects of IL1β and IL8 on enhanced proliferation in Mock-si but not MUC17-si cells (Fig. 5e). SB203580 also increased the activity of IL1β and IL8 on proliferation in T-MUC17, but not Mock cells (Fig. 5f). These results indicate that MUC17 reduces inflammatory response in GC cells, and induces cell arrest through the activation of p38 pathway.

### MUC17 interacts with myosin-9 and activates the RhoA pathway

**Fig. 6:**
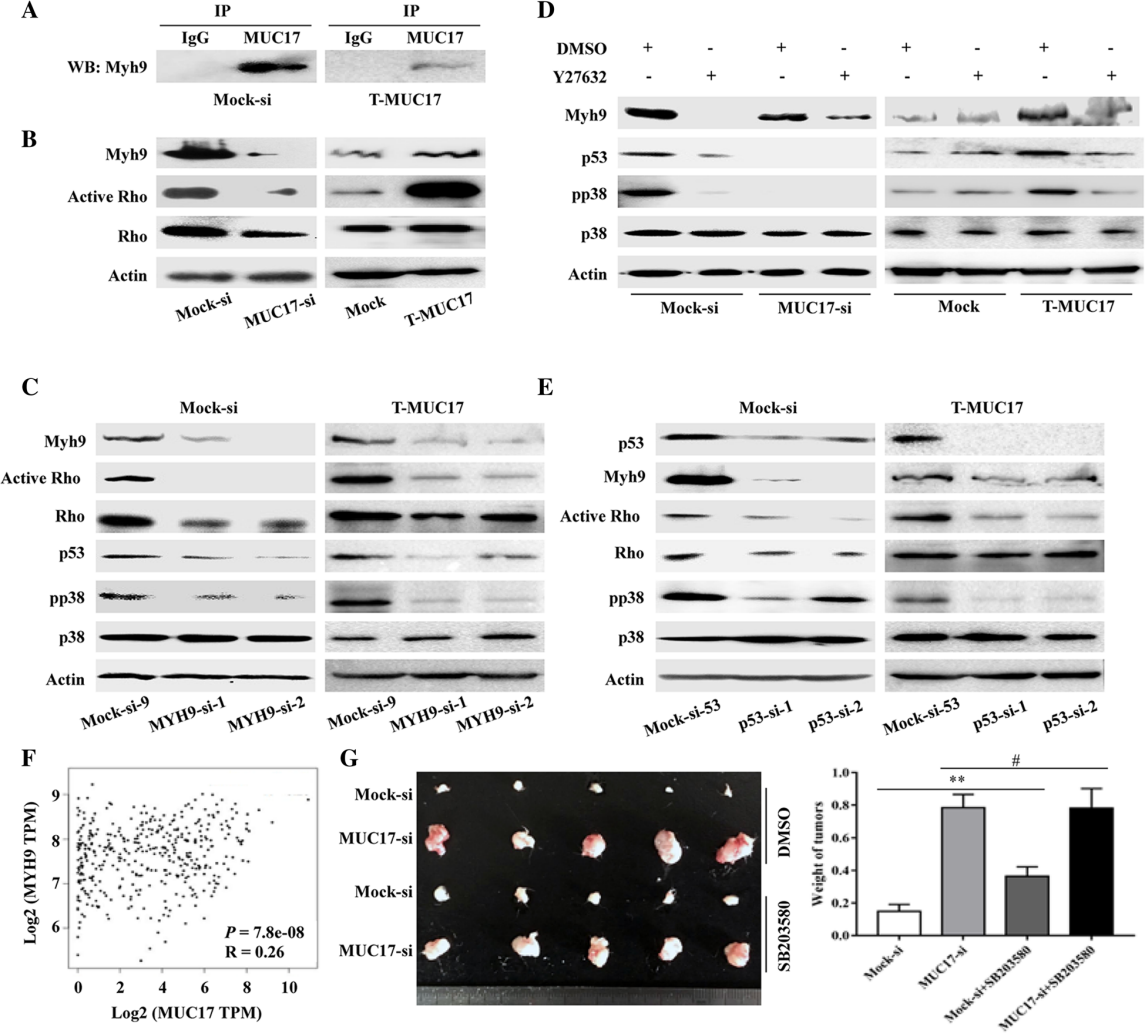
MUC17 regulates the p38 pathway by interacting with Myh9. **a** MUC17 co-immunoprecipitated with MYH9 in GC cells. Lysates prepared from Mock-si cells that endogenously expressing *MUC17* were precipitated with an anti-MUC17 antibody and the precipitants western blotted with an anti-MYH9 antibody (left). Lysates prepared from T-MUC17 cells that exogenously expressing truncated *MUC17* were precipitated with an anti-MUC17 antibody and the precipitants were blotted with an anti-MYH9 antibody (right). **b** The expression of MYH9 and activation of the RhoA pathway in *MUC17* knocked-down AGS cells and control cells (left), and in truncated MUC17 overexpressed MKN45 cells and control cells (right). **c** The effects of MYH9 on p53 expression and activation of p38 and the RhoA pathway in Mock-si (left) and T-MUC17 cells (right). **d** The effects of the RhoA pathway inhibitor Y27632 on the expression of MYH9, p53, and activation of p38 pathway in *MUC17* knocked-down AGS cells (left) and truncted MUC17 overexpressed MKN45 cells (right). **e** The effects of p53 on MYH9 expression and activation of p38 and the RhoA pathway in Mock-si (left) and T-MUC17 cells (right). **f** The expression relation between MUC17 and Myh9 by using TCGA_GC transcriptional database. **g** Left, Representative images of tumours that originated from Mock-si cells and MUC17-si cells treated with SB203580 or DMSO in Nod Scid mice. Right, Weights of tumors originated from Mock-si cells and MUC17-si cells treated with SB203580 or DMSO from 5 Nod Scid mice (#*P* > 0.05, **P < 0.001)

To elucidate the role of MUC17 in p38 pathway in GC cells, we screened for potential MUC17-interacting proteins. We performed co-immunoprecipitation using an anti-MUC17 antibody followed by LC-MS/MS assays. Myosin-9 (MYH9) was identified as a potential MUC17 interacting protein (Additional file 3: Table S7). We then immunoprecipitated Mock-si cell lysate with an anti-MUC17 antibody. Western blotting of the precipitates with an anti-MYH9 antibody indicated of MUC17 co-immunoprecipitation with MYH9 (Fig. 6a, **left**). Truncated MUC17 also interacted with MYH9 (Fig. 6a, **right**), indicating that MUC17 interacts with MYH9 through its conserved EGF domain and downstream sequence. Further, MYH9 was expressed at higher level in Mock-si cells relative to MUC17-si cells (Fig. 6b, **left**), while MYH9 level was higher in T-MUC17 cells compared to Mock cells (Fig. 6b, **right**). Thus, MUC17 regulates MYH9 expression through its EGF domain and downstream sequence.

Since MYH9 is involved in RhoA signalling, we assessed the activation state of RhoA pathway, which was more significantly activated in Mock-si cells compared to MUC17-si cells (Fig. 6b, **left**). The pathway was also more significantly activated in T-MUC17 cells compared to Mock cells (Fig. 6b, **right**). To confirm that MUC17 regulated the RhoA pathway via MYH9, we knocked down MYH9 in Mock-si cells and T-MUC17 cells using two shRNAs, MYH9-si-1 and MYH9-si-2, and a scrambled shRNA as control (Mock-si-9) (Fig. 6c). Compared to Mock-si-9 cells, the expression of the active form of RhoA was decreased in both MYH9-si-1 and MYH9-si-2 cells.

### MUC17 activates the p38 pathway and upregulates p53 expression via myosin-9 in GC cells

Our study has shown that p38 signalling is a key factor involved in MUC17-mediated inhibition of GC cell proliferation and protection against inflammatory stimulation. In Mock-si and T-MUC17 cells, we then assessed the effects of MYH9 on p38 signalling. We found that the expression of pp38 was downregulated in MYH9-si-1 and MYH9-si-2 cells compared to Mock-si-9 cells (Fig. 6c), suggesting that MUC17 activates the p38 pathway via MYH9. However, the MUC17-induced increasing in p53 expression in GC cells was not linked to activation of the p38 pathway (Fig. 5b), we therefore examined the effect of MYH9 on p53 expression. Compared to Mock-si-9 cells, the p53 expression was lower in both MYH9-si-1 and MYH9-si-2 GC cells (Fig. 6c). Thus, the effect of MUC17 on activation of the p38 pathway and increasing expression of p53 in GC cells were mediated via MYH9.

### MUC17 upregulates the expression of MYH9 and p53, and activates the p38 pathway in GC cells through RhoA signalling

Other study showed that RhoA signalling regulates MYH9 expression in GC cells, suggesting a link between RhoA signalling and MYH9 expression. We examined this possibility using a RhoA pathway inhibitor, Y27632. In Mock-si GC cells, Y27632 treatment decreased MYH9 expression compared to control cells treated with DMSO (Fig. 6d, **left**). Similarly, Y27632 treatment of T-MUC17 cells also decreased MYH9 expression compared to control cells treated with DMSO (Fig. 6d, **right**).

As RhoA signalling was found to induce MYH9 expression in GC cells, the effects of active RhoA on the p38 pathway and p53 expression were then examined. In Mock-si cells, the expression levels of pp38 and p53 were both reduced after Y27632 treatment compared to cells treated with DMSO (Fig. 6d, **left**). The level of pp38 and p53 were also lower in T-MUC17 cells treated with Y27632 compared to cells treated with DMSO (Fig. 6d, **right**). Thus MUC17 activates the p38 pathway and upregulates p53 through the RhoA signalling cascade.

### MUC17 activates the p38 pathway through a MYH9-RhoA-p53 regulatory feedback loop

**Fig. 7:**
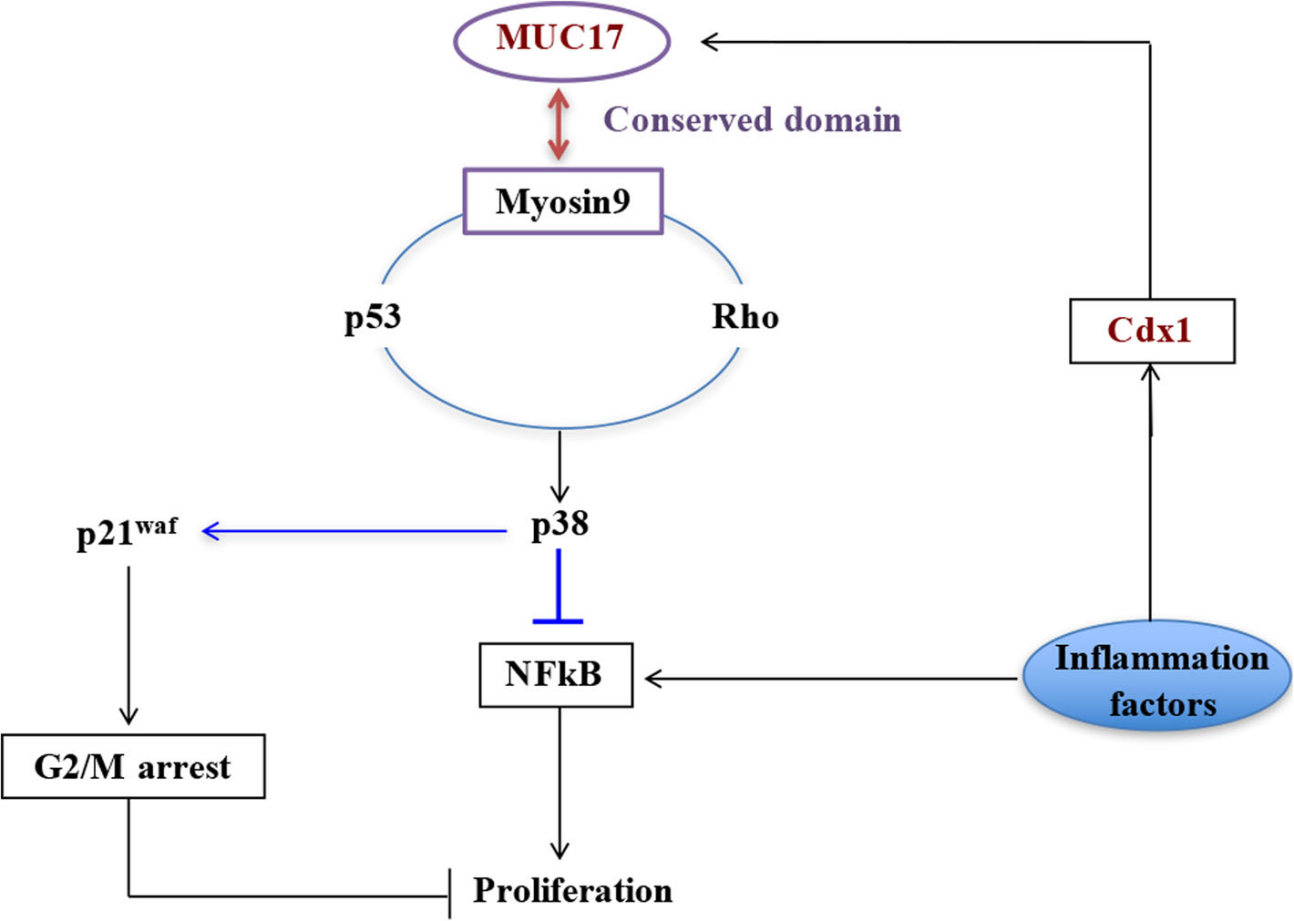
Schematic of MUC17 inhibits the progression of human gastric cancer by limiting inflammatory responses. Inflammatory factors induce the expression of MUC17 through upregulating CDX1. MUC17 is dependent on its conserved epidermal growth factor domain and on downstream sequences to enable its interaction with myosin-9, resulting in a sustained regulatory feedback loop between myosin-9, p53, and RhoA, and then activation of p38 to negatively regulate the NFκB pathway in GC cells. Inflammatory factor promotes cancer cell growth through activating NFκB pathway, hence, MUC17 can limiting inflammatory responses

As both the RhoA pathway and MYH9 were found to promote p53 expression, we investigated whether a reverse relationship existed and whether p53 regulated RhoA signalling or MYH9 expression. p53 then was knocked-down in Mock-si and T-MUC17 cells using two shRNAs, p53-si-1 and p53-si-2, and a scrambled shRNA as a control (Mock-si-53) (Fig. 6e). Compared to Mock-si-53 cells, the expression of pp38, the active form of RhoA, and MYH9 was each decreased in the p53-si-1 and p53-si-2 cells. These data suggest that a regulatory feedback loop linking between MYH9, p53, and RhoA pathway, with MUC17 as stabilize of the loop (Fig. 7).

### The relation between MUC17, Myh9 and p38 pathway in vivo

To provide the mechanistic insight for the link between MUC17, inflammation and tumorigenesis in vivo, we detected the expression relation between MUC17 and Myh9 by using TCGA_GC transcriptional database. As shown in Fig. 6f, MUC17 expression was positive correlated with Myh9 expression, indicating the mechanism detected in human GC cell lines also exist in vivo. We further performed the xenograft using GC sh-control and sh-MUC17 cells and locally treating the tumors with p38 inhibitor to confirm the relation between MUC17 and p38 in vivo. As shown in Fig. 6g, the volume of xenograft tumors originated from Mock-si cells were smaller than xenograft tumors originated from MUC17-si cells. After treating the tumors originated from Mock-si cells with p38 inhibitor, the volume of tumors was significantly increased.

## Discussion

Many genes are found to be highly expressed in cancer cells and some are related to improved prognosis. For example, CXCL16 expression was consistently up-regulated in colorectal cancer tissues than in normal mucosa, and the high CXCL16 expression patients showed significantly better prognosis than the low expression patients [10]. OCIAD2 was highly expressed in adenocarcinoma mixed subtype with bronchioloalveolar carcinoma component and was associated with improved prognosis [11]. It should be noted that the feature of these genes is different from that of oncogenes, which are highly expressed in tumour cells and are associated with poor prognosis, and that of tumour suppressor genes are generally expressed at lower level in tumour cells and are associated with favourable prognosis. However, the mechanism underlying this association is not clear.

In this study, we revealed that MUC17 serves as a response protein, and protects gastric epithelial cells from harmful insults, such as inflammation. Through the conserved EGF domain and downstream sequence, MUC17 interacts with MYH9 and subsequently is involved in the activation of the p38 pathway via a positive regulatory feedback loop in the MYH9, p53, and RhoA pathway. This leads to the inactivation of the NFκB pathway. Inflammatory cytokines stimulate the accumulation of MUC17 in GC cells via upregulation of CDX1 transcription factor. This leads to increased expression of MUC17 in cancer cells, thereby inhibiting cancer cell proliferation and improving survival of patients with GC (Fig. 7).

Through bioinformatics analysis, we found that the promoter of CDX1 comprises of CpG islands, indicating that the expression of CDX1 may be regulated by methylation. Wong et.al showed that the methylation of *CDX1* induced the low level of *CDX1* expression in some colorectal cancer cell lines [12]. The epigenetic therapies have been tested in early-stage clinical trials in patients with relapsed and chemorefractory cancers. Some of these patients have achieved complete responses [13]. We therefore propose that the epigenetic therapies may prolong the survival of patients with GC by upregulating the expression of CDX1.

Our data showed that MUC17 interacted with Myh9, which regulated the expression of p53, this phenomenon was also observed by Schramek et al. They unveiled Myh9 as a tumor suppressor though regulating the posttranscriptional p53 stabilization in squamous cell carcinomas [14]. Our data indicate that MUC17 stabilizes the regulatory feedback loop in MYH9, p53, and the RhoA pathways. Li et al. reported that Ubr3 negatively regulates the mono-ubiquitination of MYH9, hence, we propose that MUC17 protects MYH9 from degradation by masking its ubiquitinations sites [15]. Future studies will clarify the mechanisms by which MUC17 increases MYH9 expression to confer GC cells with decreased malignancy.

## Conclusion

The expression of MUC17 was induced by inflammatory factor through CDX1. However, as a protect factor, MUC17 inhibited the role of inflammatory factor through sustaining the MYH9-p53-RhoA regulatory feedback loop, then activating p38 signalling to inactivate NFkB pathway. The results of this study provide a new basis for studying the mechanism of gene that highly expressed in cancer but associated with better prognosis. Cumulatively, our results suggest that MUC17 is a GC biomarker (including early stage of GC) which highlight its potential clinical utility as a promising diagnostic and therapeutic target of GC.

## Additional files

Additional file 1: **Figure S1** The differential expression of *MUC1*, *MUC12*, *MUC13*, and *MUC16* in GC and normal tissues in the TCGA_GC cohort. (JPG 108 kb)

Additional file 2: **Figure S2** Analysis the expression relation between MUC1, MUC12, MUC13, MUC16 and IL1β/IL8 by using TCGA_GC transcriptional database. (JPG 183 kb)

Additional file 3: **Table S1** The primers and sequences used in this study. **Table S2** The clinical characteristics of the patients with GC. **Table S3** The differential expression of MUC17 in GC tissues and normal tissues. **Table S4** Univariate and multivariate analysis of clinicopathological features and MUC17 expression in GC. **Table S5** The differential expression of MUC17 in early stage GC tissues and normal tissues. **Table S6** The clinical characteristics of the patients with early stage GC. **Table S7** Potential proteins interacted with MUC17 (DOCX 77 kb)

## Data Availability

The dataset(s) supporting the findings of this study are included within the article.

## Abbreviations

ChIP: Immunoprecipitation
EMSA: Electrophoretic mobility shift assay
GC: Gastric cancer
IHC: Immunohistochemistry
MUC17: Mucin17
TCGA: The Cancer Genome Atlas
