# Supplementary figures and images for: Mucin 17 inhibits the progression of human gastric cancer by limiting inflammatory responses through a MYH9-p53-RhoA regulatory feedback loop
Source: J Exp Clin Cancer Res. 2019 Jul 1;38:283. doi: 10.1186/s13046-019-1279-8 (PMC6604468; doi:10.1186/s13046-019-1279-8)

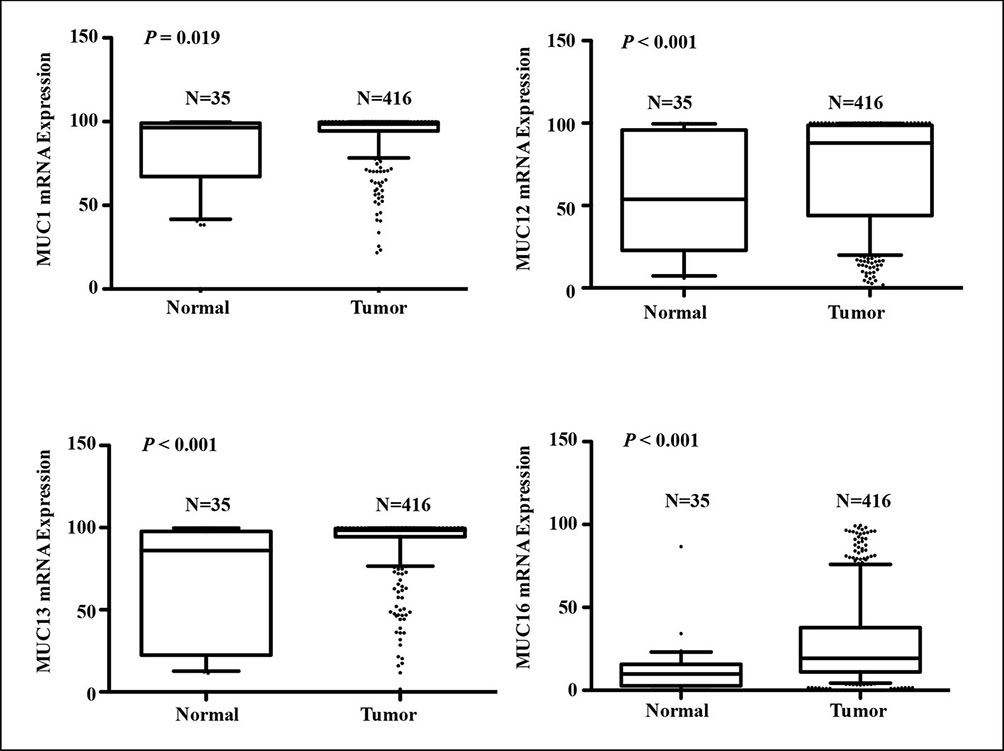

Supplement: Supplementary file 1 — Figure S1 The differential expression of MUC1, MUC12, MUC13, and MUC16 in GC and normal tissues in the TCGA_GC cohort. (JPG 108 kb) [file 13046_2019_1279_MOESM1_ESM.jpg]

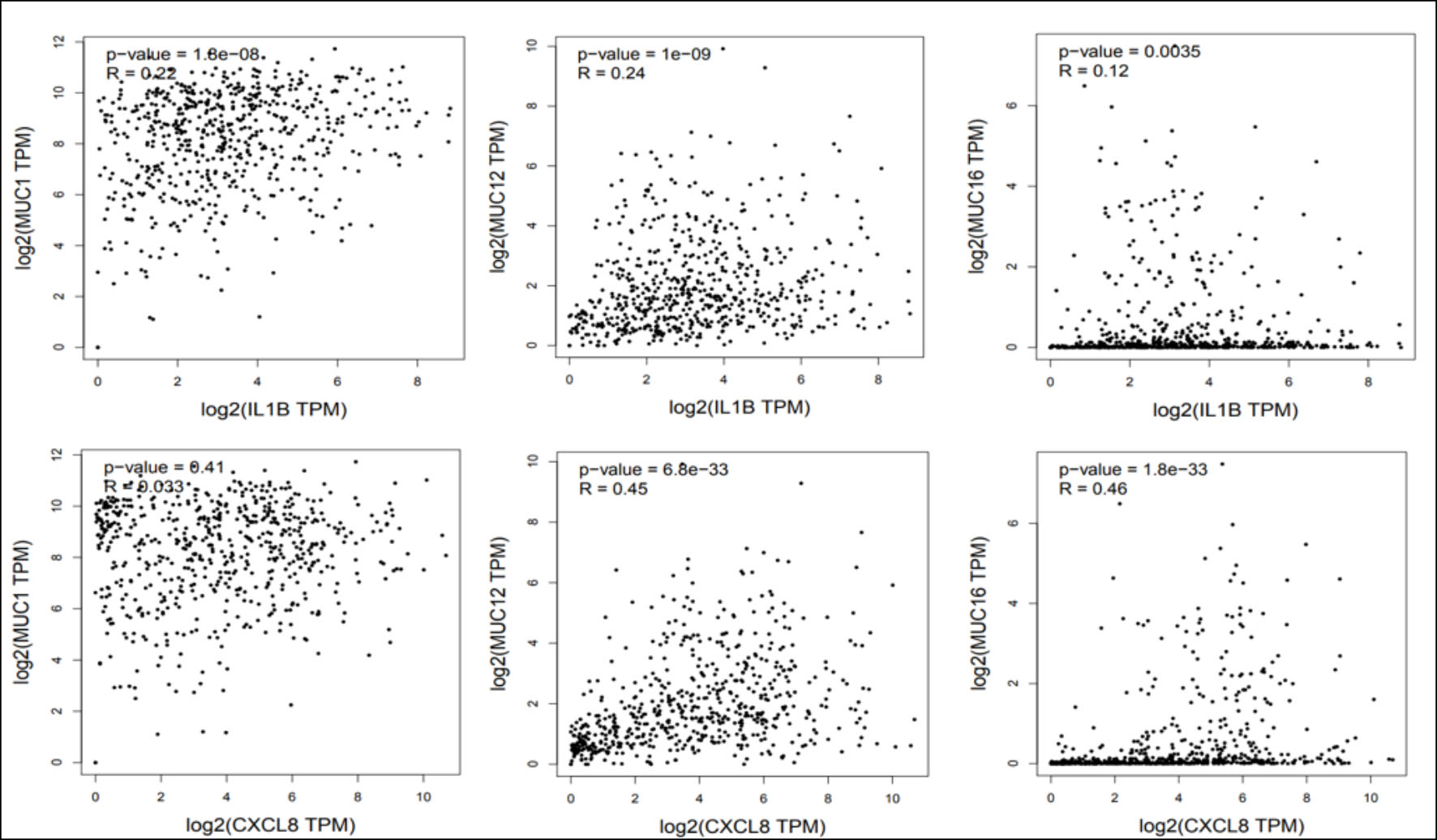

Supplement: Supplementary file 2 — Figure S2 Analysis the expression relation between MUC1, MUC12, MUC13, MUC16 and IL1β/IL8 by using TCGA_GC transcriptional database. (JPG 183 kb) [file 13046_2019_1279_MOESM2_ESM.jpg]
