# Supplementary material for: Mucin 17 inhibits the progression of human gastric cancer by limiting inflammatory responses through a MYH9-p53-RhoA regulatory feedback loop
Source: J Exp Clin Cancer Res. 2019 Jul 1;38:283. doi: 10.1186/s13046-019-1279-8 (PMC6604468; doi:10.1186/s13046-019-1279-8)
Supplement: Supplementary file 3 — Table S1 The primers and sequences used in this study. Table S2 The clinical characteristics of the patients with GC. Table S3 The differential expression of MUC17 in GC tissues and normal tissues. Table S4 Univariate and multivariate analysis of clinicopathological features and MUC17 expression in GC. Table S5 The differential expression of MUC17 in early stage GC tissues and normal tissues. Table S6 The clinical characteristics of the patients with early stage GC. Table S7 Potential proteins interacted with MUC17 (DOCX 77 kb) [file 13046_2019_1279_MOESM3_ESM.docx]

Table S1．The primers and sequences used in this study**.**

| **Gene** | **Experiment** | **Forward** | **Reverse** |
| --- | --- | --- | --- |
| **MUC17** | Realtime-PCR | 5’-GGGCCAGCATAGCTTCGA-3’ | 5’-GCTACAGGAATTGTGGGAGTTGA-3’ |
| **CDX1** | Realtime-PCR | 5’-TGAACGGCAGGTGAAGATCTG-3’ | 5’-GCTGTTTCTTCTTGTTCACTTTGC-3’ |
| **ACTIN** | Realtime-PCR | 5’-TTAGTTGCGTTACACCCTTTC-3’ | 5’-ACCTTCACCGTTCCAGTTT-3’ |
| **FA** | Plasmid construct | 5’-TCCCCCGGGTtattttatttatttatt-3’ | 5’-CCGCTCGAGCCTGTTCTGCAGCAGCTT-3’ |
| **FB** | Plasmid construct | 5’-TCCCCCGGGTtcatcatgttggccagg-3’ | 5’-CCGCTCGAGCCTGTTCTGCAGCAGCTT-3’ |
| **FC** | Plasmid construct | 5’-TCCCCCGGGCagtccctcttgcccatc-3’ | 5’-CCGCTCGAGCCTGTTCTGCAGCAGCTT-3’ |
| **WT**  **M1**  **M2** | EMSA  EMSA  EMSA | 5’-gggTtattttatttatttatttt-3’  5’-gggTtattttaGGGatttatttt-3’  5’-gggTtattttatttaGGGaGGtt-3’ | 5’-aaaataaataaataaaataaccc-3’  5’-aaaataaatCCCtaaaataaccc-3’  5’-aaCCtCCCtaaataaaataaccc-3’ |
| **MUC17** | ShRNAi | ATACCAACCTCGACTCTTA |  |
| **CDX1** | ShRNAi | GACTCGGACCAAGGACAAG |  |
| **MYH9-1** | ShRNAi | CGGCAAATTCATTCGCATCAA |  |
| **MYH9-2** | ShRNAi | GCGTTACTACTCAGGGCTCAT |  |
| **p53-1**  **p53-2** | ShRNAi  ShRNAi | GTCCAGATGAAGCTCCCAGAA  AGGAAATTTGCGTGTGGAGTA |  |

Table S2．The clinical characteristics of the patients with GC.

| **Sample** | **Gender** | **Age** | **Differentiation degree** | **TNM** | **Treatment prior to surgery** | **Survive** | **Time (Days)** |
| --- | --- | --- | --- | --- | --- | --- | --- |
| GC-01 | Male | 67 | Low | T4N1M0 | No treatment | Dead | 180 |
| GC-02 | Male | 43 | Low | T2N0M0 | No treatment | Live | 1300 |
| GC-03 | Male | 61 | Low | T3N2M0 | No treatment | Dead | 90 |
| GC-04 | Male | 50 | Low | T3N1M0 | No treatment | Live | 1300 |
| GC-05 | Female | 47 | Low | T3N1M0 | No treatment | Live | 1300 |
| GC-06 | Male | 67 | Median | T3N0M0 | No treatment | Live | 1300 |
| GC-07 | Female | 65 | Low | T3N2M0 | No treatment | Dead | 330 |
| GC-08 | Male | 58 | Median | T2N1M0 | No treatment | Dead | 1080 |
| GC-09 | Male | 65 | Low | T3N1M0 | No treatment | Dead | 1080 |
| GC-10 | Male | 53 | Low | T3N0M0 | No treatment | Live | 1300 |
| GC-11 | Male | 65 | Median | T3N1M0 | No treatment | Dead | 480 |
| GC-12 | Male | 51 | Low | T3N1M0 | No treatment | Live | 1300 |
| GC-13 | Male | 61 | Median | T3N1M0 | No treatment | Live | 1300 |
| GC-14 | Male | 46 | Median | T3N2M0 | No treatment | Dead | 330 |
| GC-15 | Male | 56 | Low | T2N0M0 | No treatment | Dead | 330 |
| GC-16 | Male | 62 | Median | T2N1M0 | No treatment | Dead | 1110 |
| GC-17 | Male | 58 | Median | T2N1M0 | No treatment | Live | 1300 |
| GC-18 | Male | 53 | Median | T2N2M0 | No treatment | Dead | 870 |
| GC-19 | Male | 68 | Median | T2N1M0 | No treatment | Live | 1300 |
| GC-20 | Female | 66 | Median | T2N0M0 | No treatment | Live | 1300 |
| GC-21 | Female | 65 | Median | T3N1M0 | No treatment | Dead | 1080 |
| GC-22 | Male | 67 | Median | T3N2M0 | No treatment | Dead | 150 |
| GC-23 | Male | 59 | Median | T3N1M0 | No treatment | Live | 1300 |
| GC-24 | Male | 60 | Median | T3N1M0 | No treatment | Live | 1300 |
| GC-25 | Male | 53 | Median | T3N0M0 | No treatment | Live | 1300 |
| GC-26 | Male | 62 | Median | T2N0M0 | No treatment | Live | 1300 |
| GC-27 | Male | 53 | Low | T2N1M0 | No treatment | Dead | 570 |
| GC-28 | Male | 58 | Median | T3N1M0 | No treatment | Dead | 330 |
| GC-29 | Male | 61 | Median | T2N2M0 | No treatment | Dead | 270 |
| GC-30 | Male | 54 | Low | T2N0M0 | No treatment | Live | 1300 |
| GC-31 | Male | 50 | Low | T3N1M0 | No treatment | Live | 1300 |
| GC-32 | Male | 68 | Low | T3N0M0 | No treatment | Live | 1300 |
| GC-33 | Male | 53 | Low | T2N0M0 | No treatment | Dead | 1300 |
| GC-34 | Male | 68 | NA | T2N1M0 | No treatment | Dead | 240 |
| GC-35 | Male | 56 | Median | T4N0M0 | No treatment | Dead | 660 |
| GC-36 | Female | 65 | Low | T3N1M0 | No treatment | Dead | 930 |
| GC-37 | Male | 65 | NA | T2N1M0 | No treatment | Live | 1300 |
| GC-38 | Male | 69 | Median | T3N2M0 | No treatment | Dead | 840 |
| GC-39 | Male | 49 | Low | T3N3M0 | No treatment | Dead | 300 |
| GC-40 | Male | 69 | Low | T2N1M0 | No treatment | Dead | 150 |
| GC-41 | Male | 49 | Median | T2N2M0 | No treatment | Live | 1300 |
| GC-42 | Male | 56 | Median | T2N0M0 | No treatment | Live | 1300 |
| GC-43 | Female | 52 | Median | T2N2M0 | No treatment | Live | 1300 |
| GC-44 | Male | 60 | Median | T2N2M0 | No treatment | Live | 1300 |
| GC-45 | Male | 54 | Median | T2N1M0 | No treatment | Dead | 750 |
| GC-46 | Male | 64 | Median | T4N2M0 | No treatment | Dead | 360 |
| GC-47 | Male | 52 | Median | T2N0M0 | No treatment | Live | 1300 |
| GC-48 | Male | 69 | Median | T3N0M0 | No treatment | Live | 1300 |
| GC-49 | Male | 53 | Median | T3N2M0 | No treatment | Dead | 1080 |
| GC-50 | Male | 62 | Median | T2N1M0 | No treatment | Dead | 840 |
| GC-51 | Male | 66 | Low | T3N3M0 | No treatment | Dead | 90 |
| GC-52 | Male | 63 | Median | T2N0M0 | No treatment | Live | 1300 |
| GC-53 | Male | 50 | Median | T2N0M0 | No treatment | Live | 1300 |
| GC-54 | Male | 67 | Median | T2N1M0 | No treatment | Dead | 60 |
| GC-55 | Male | 68 | Median | T2N0M0 | No treatment | Live | 1300 |
| **Sample** | **Gender** | **Age** | **Differentiation degree** | **TNM** | **Treatment prior to surgery** | **Survive** | **Time (Days)** |
| GC-56 | Male | 57 | Median | T2N0M0 | No treatment | Live | 1300 |
| GC-57 | Male | 59 | Median | T2N0M0 | No treatment | Live | 1300 |
| GC-58 | Male | 48 | Median | T3N1M1 | No treatment | Dead | 720 |
| GC-59 | Male | 57 | Median | T2N1M0 | No treatment | Dead | 510 |
| GC-60 | Male | 65 | Median | T3N1M0 | No treatment | Dead | 600 |
| GC-61 | Female | 66 | Median | T3N3M0 | No treatment | Dead | 930 |
| GC-62 | Male | 63 | Low | T3N1M0 | No treatment | Live | 1300 |
| GC-63 | Male | 56 | Median | T2N2M0 | No treatment | Live | 1300 |
| GC-64 | Male | 65 | Median | T3N3M0 | No treatment | Live | 1300 |
| GC-65 | Male | 58 | Median | T3N2M0 | No treatment | Live | 1300 |
| GC-66 | Male | 50 | Low | T3N1M0 | No treatment | Dead | 360 |
| GC-67 | Male | 69 | Median | T3N2M0 | No treatment | Dead | 300 |
| GC-68 | Female | 77 | Median | T2N1M0 | No treatment | Live | 1300 |
| GC-69 | Male | 68 | Median | T2N0M0 | No treatment | Live | 1300 |
| GC-70 | Male | 58 | Low | T2N3M0 | No treatment | Dead | 780 |
| GC-71 | Male | 67 | Median | T2N2M0 | No treatment | Live | 1300 |
| GC-72 | Male | 69 | Median | T2N0M0 | No treatment | Live | 1300 |
| GC-73 | Male | 57 | Median | T2N2M0 | No treatment | Live | 1300 |
| GC-74 | Male | 66 | Median | T2N1M0 | No treatment | Dead | 600 |
| GC-75 | Male | 50 | Low | T3N2M0 | No treatment | Dead | 660 |
| GC-76 | Female | 69 | Median | T3N2M0 | No treatment | Dead | 450 |
| GC-77 | Male | 50 | Median | T3N2M0 | No treatment | Live | 1300 |
| GC-78 | Male | 64 | Median | T2N3M0 | No treatment | Live | 1300 |
| GC-79 | Male | 53 | Median | T2N2M0 | No treatment | Live | 1300 |
| GC-80 | Male | 67 |  | T2N1M0 | No treatment | Dead | 750 |
| GC-81 | Male | 67 | Median | T2N0M0 | No treatment | Live | 1300 |
| GC-82 | Female | 69 | Median | T2N0M0 | No treatment | Live | 1300 |
| GC-83 | Male | 48 | Median | T2N2M0 | No treatment | Live | 1300 |
| GC-84 | Female | 64 | Low | T1N0M0 | No treatment | Live | 1300 |
| GC-85 | Male | 55 | Median | T3N3M0 | No treatment | Live | 1300 |
| GC-86 | Male | 67 | Median | T3N0M0 | No treatment | Live | 1300 |
| GC-87 | Female | 66 | Median | T2N0M0 | No treatment | Dead | 210 |
| GC-88 | Male | 64 | Median | T2N0M0 | No treatment | Live | 1300 |
| GC-89 | Male | 66 | Median | T2N0M0 | No treatment | Live | 1300 |
| GC-90 | Male | 62 | Median | T2N2M0 | No treatment | Dead | 690 |
| GC-91 | Male | 68 | Low | T3N2M0 | No treatment | Dead | 690 |
| GC-92 | Male | 49 | Low | T3N0M0 | No treatment | Dead | 720 |
| GC-93 | Male | 59 | Median | T1N0M0 | No treatment | Live | 1300 |
| GC-94 | Male | 50 | Median | T2N0M0 | No treatment | Live | 1300 |
| GC-95 | Male | 68 | Median | T3N1M0 | No treatment | Dead | 810 |
| GC-96 | Female | 56 | Low | T2N3M0 | No treatment | Dead | 150 |
| GC-97 | Male | 53 | Low | T2N0M0 | No treatment | Live | 1300 |
| GC-98 | Female | 73 | Median | T2N1M0 | No treatment | Live | 1300 |
| GC-99 | Male | 60 | Median | T3N0M0 | No treatment | Dead | 600 |
| GC-100 | Male | 66 | Median | T3N2M1 | No treatment | Dead | 240 |
| GC-101 | Male | 61 | Median | T3N0M0 | No treatment | Live | 1300 |
| GC-102 | Female | 71 | Median | T1N0M0 | No treatment | Live | 1300 |
| GC-103 | Male | 76 | Median | T2N1M0 | No treatment | Live | 1300 |
| GC-104 | Male | 63 | Median | T2N1M0 | No treatment | Dead | 0 |
| GC-105 | Male | 42 | Median | T3N0M0 | No treatment | Live | 1300 |
| GC-106 | Male | 52 | Median | T2N0M0 | No treatment | Live | 1300 |
| GC-107 | Male | 70 | Median |  | No treatment | Dead | 30 |
| GC-108 | Male | 66 | Median | T3N1M0 | No treatment | Dead | 0 |
| GC-109 | Male | 61 |  | T1N0M0 | No treatment | Live | 1300 |
| GC-110 | Male | 63 | Median | T1N0M0 | No treatment | Dead | 1080 |
| GC-111 | Male | 69 | Median | T3N1M0 | No treatment | Dead | 300 |
| GC-112 | Male | 56 | Median | T1N0M0 | No treatment | Live | 1300 |
| GC-113 | Male | 64 | Median | T1N0M1 | No treatment | Dead | 180 |
| **Sample** | **Gender** | **Age** | **Differentiation degree** | **TNM** | **Treatment prior to surgery** | **Survive** | **Time (Days)** |
| GC-114 | Female | 57 | Median | T3N2M0 | No treatment | Live | 1300 |
| GC-115 | Male | 63 | Median | T3N2M0 | No treatment | Live | 1300 |
| GC-116 | Male | 83 | Median | T3N2M0 | No treatment | Dead | 480 |
| GC-117 | Male | 19 | Median | T2N0M0 | No treatment | Live | 1300 |
| GC-118 | Female | 46 | Low | T4N1M0 | No treatment | Dead | 270 |
| GC-119 | Female | 71 | Low | T4N2M1 | No treatment | Dead | 150 |
| GC-120 | Female | 72 | Low | T3N0M0 | No treatment | Live | 1300 |
| GC-121 | Female | 66 | Low | T2N1M0 | No treatment | Dead | 1050 |
| GC-122 | Female | 58 | Low | T3N1M0 | No treatment | Dead | 180 |
| GC-123 | Male | 61 | Low | T3N0M0 | No treatment | Live | 1300 |
| GC-124 | Male | 42 | Low | T1N0M0 | No treatment | Live | 1300 |
| GC-125 | Female | 75 | Low | T4N2M0 | No treatment | Dead | 300 |
| GC-126 | Female | 43 | Low | T2N0M0 | No treatment | Live | 1300 |
| GC-127 | Male | 61 | Low | T2N0M0 | No treatment | Live | 1300 |
| GC-128 | Male | 68 | Low | T2N0M0 | No treatment | Live | 1300 |
| GC-129 | Male | 59 | Low | T4N1M1 | No treatment | Live | 1300 |
| GC-130 | Female | 47 | Low | T3N1M0 | No treatment | Dead | 930 |
| GC-131 | Male | 45 | Median |  | No treatment | Dead | 120 |
| GC-132 | Female | 51 | Low | T4N2M1 | No treatment | Dead | 450 |
| GC-133 | Male | 82 | Low | T3N1M0 | No treatment | Live | 1300 |
| GC-134 | Female | 84 | Low | T3N1M0 | No treatment | Live | 1300 |
| GC-135 | Male | 69 | Low | T3N1M0 | No treatment | Live | 1300 |
| GC-136 | Male | 75 | Low | T3N1M0 | No treatment | Dead | 390 |
| GC-137 | Male | 63 | Low | T3N0M0 | No treatment | Dead | 390 |
| GC-138 | Male | 48 | Low | T3N2M0 | No treatment | Dead | 450 |
| GC-139 | Male | 64 | Low | T3N1M0 | No treatment | Dead | 780 |
| GC-140 | Male | 67 | Low | T3N2M0 | No treatment | Dead | 120 |
| GC-141 | Male | 74 | Low | T3N1M0 | No treatment | Dead | 30 |
| GC-142 | Male | 66 | Low | T3N0M0 | No treatment | Live | 1300 |
| GC-143 | Male | 74 | Low | T3N1M0 | No treatment | Dead | 60 |
| GC-144 | Male | 67 | Low | T3N1M0 | No treatment | Dead | 360 |
| GC-145 | Male | 50 | Low | T3N0M0 | No treatment | Dead | 90 |
| GC-146 | Male | 70 | Median | T4N2M0 | No treatment | Dead | 1050 |
| GC-147 | Male | 74 | Median | T3N0M0 | No treatment | Live | 1300 |
| GC-148 | Female | 76 | Low | T3N2M0 | No treatment | Dead | 810 |
| GC-149 | Male | 48 | Median | T3N1M0 | No treatment | Live | 1110 |
| GC-150 | Female | 54 | Low | T4N0M0 | No treatment | Live | 1300 |
| GC-151 | Male | 47 | Median | T3N0M0 | No treatment | Live | 1300 |
| GC-152 | Male | 67 | Low | T3N0M0 | No treatment | Live | 1300 |
| GC-153 | Male | 69 | Median | T3N1M0 | No treatment | Live | 1300 |
| GC-154 | Male | 64 | Median | T2N1M0 | No treatment | Dead | 630 |
| GC-155 | Male | 57 | Low | T3N1M0 | No treatment | Dead | 660 |
| GC-156 | Male | 75 | Median | T3N2M0 | No treatment | Dead | 240 |
| GC-157 | Male | 75 | Low | T3N1M0 | No treatment | Dead | 1050 |
| GC-158 | Female | 60 | Low | T3N2M0 | No treatment | Live | 1300 |
| GC-159 | Male | 66 | Median | T3N0M0 | No treatment | Live | 750 |
| GC-160 | Male | 65 |  | T3N2M0 | No treatment | Dead | 360 |
| GC-161 | Male | 73 | Low | T2N2M0 | No treatment | Dead | 360 |
| GC-162 | Male | 78 | Median | T3N0M0 | No treatment | Dead | 870 |
| GC-163 | Male | 79 | Median | T2N0M0 | No treatment | Dead | 450 |

**Table S3．The differential expression of MUC17 in GC tissues and normal tissues.**

|  | **Expression** | | |  |  |
| --- | --- | --- | --- | --- | --- |
|  | **Low** | **Media** | **High** |  |  |
| **Normal** | 42 | 9 | 0 |  |  |
| **Gastric cancer** | 59 | 66 | 38 |  |  |
| ***P* value** |  |  |  |  | <0.001 |

**Table S4．Univariate and multivariate analysis of clinicopathological features and MUC17 expression in GC**

|  | **Univariate** | | |  | **Multivariate** | | |
| --- | --- | --- | --- | --- | --- | --- | --- |
|  | **HR** | **95%CI** | ***P* value** |  | **HR** | **95%CI** | ***P* value** |
| Sex | 0.035 | 0.591 to 1.815 | 0.902 |  | -0.222 | 0.443 to 1.448 | 0.462 |
| Age ≥60 year | 0.531 | 1.071 to 2.700 | **0.024** |  | 0.697 | 1.220 to 3.305 | **0.006** |
| Differentiation degree | 0.240 | 1.016 to 1.591 | **0.036** |  | 0.257 | 1.005 to 1.665 | **0.045** |
| Tumor invasive depth | 0.587 | 1.280 to 2.528 | **0.001** |  | 0.294 | 0.888 to 2.029 | 0.162 |
| Lymph node metastasis | 0.521 | 1.341 to 2.113 | **<0.001** |  | 0.563 | 1.371 to 2.247 | **<0.001** |
| Distant metastasis | 1.131 | 1.246 to 7.710 | **0.015** |  | 0.843 | 0.821 to 6.579 | 0.112 |
| MUC17 expression | -0.375 | 0.516 to 0.914 | **0.010** |  | -0.395 | 0.489 to 0.929 | **0.016** |

**Table S5．The differential expression of MUC17 in early stage GC tissues and normal tissues.**

|  | **Expression** | | |  |  |
| --- | --- | --- | --- | --- | --- |
|  | **Low** | **Media** | **High** |  |  |
| **Normal** | 37 | 3 | 0 |  |  |
| **Early stage GC** | 20 | 11 | 11 |  |  |
| ***P* value** |  |  |  |  | <0.001 |

**Table S6．The clinical characteristics of the patients with early stage GC.**

| **Sample** | **Sex** | **Age** | **Differentiation degree** | **TNM** | **Treatment prior to surgery** |
| --- | --- | --- | --- | --- | --- |
| EGC-01 | Male | 50 | High | T1N1M0 | No treatment |
| EGC-02 | Male | 61 | High | T1N0M0 | No treatment |
| EGC-03 | Male | 54 | High | T1N1M0 | No treatment |
| EGC-04 | Male | 45 | High | T2N0M0 | No treatment |
| EGC-05 | Female | 40 | High | T1N1M0 | No treatment |
| EGC-06 | Male | 47 | High | T1N1M0 | No treatment |
| EGC-07 | Female | 49 | High | T1N1M0 | No treatment |
| EGC-08 | Male | 63 | High | T1N1M0 | No treatment |
| EGC-09 | Male | 71 | High | T1N1M0 | No treatment |
| EGC-10 | Male | 68 | High | T2N0M0 | No treatment |
| EGC-11 | Male | 69 | High | T1N1M0 | No treatment |
| EGC-12 | Male | 60 | High | T1N1M0 | No treatment |
| EGC-13 | Male | 47 | High | T1N1M0 | No treatment |
| EGC-14 | Male | 65 | High | T1N1M0 | No treatment |
| EGC-15 | Female | 51 | High | T1N1M0 | No treatment |
| EGC-16 | Female | 68 | High | T1N1M0 | No treatment |
| EGC-17 | Female | 66 | High | T2N0M0 | No treatment |
| EGC-18 | Male | 59 | High | T2N0M0 | No treatment |
| EGC-19 | Male | 61 | High | T2N0M0 | No treatment |
| EGC-20 | Female | 50 | High | T2N0M0 | No treatment |
| EGC-21 | Female | 52 | High | T2N0M0 | No treatment |
| EGC-22 | Male | 65 | High | T1N1M0 | No treatment |
| EGC-23 | Male | 68 | High | T1N1M0 | No treatment |
| EGC-24 | Male | 57 | High | T1N1M0 | No treatment |
| EGC-25 | Male | 50 | High | T1N1M0 | No treatment |
| EGC-26 | Male | 53 | High | T1N1M0 | No treatment |
| EGC-27 | Male | 69 | High | T2N0M0 | No treatment |
| EGC-28 | Male | 48 | High | T1N1M0 | No treatment |
| EGC-29 | Male | 64 | High | T1N1M0 | No treatment |
| EGC-30 | Male | 50 | High | T1N1M0 | No treatment |
| EGC-31 | Male | 68 | High | T1N1M0 | No treatment |
| EGC-32 | Male | 57 | High | T1N1M0 | No treatment |
| EGC-33 | Male | 59 | High | T1N1M0 | No treatment |
| EGC-34 | Male | 65 | High | T1N1M0 | No treatment |
| EGC-35 | Male | 63 | High | T2N0M0 | No treatment |
| EGC-36 | Female | 58 | High | T1N1M0 | No treatment |
| EGC-37 | Male | 58 | High | T1N1M0 | No treatment |
| EGC-38 | Male | 50 | High | T1N1M0 | No treatment |
| EGC-39 | Male | 64 | High | T1N1M0 | No treatment |
| EGC-40 | Male | 67 | High | T2N0M0 | No treatment |
| EGC-41 | Male | 67 | High | T1N1M0 | No treatment |
| EGC-42 | Male | 55 | High | T1N1M0 | No treatment |

**Table S7. Potential proteins interacted with MUC17.**

| Protein | Number of peptides | Score |
| --- | --- | --- |
| MYH9 | 76 | 11003 |
| KI-67 | 3 | 88 |
| ACINU | 4 | 84 |
| TOP2B | 4 | 83 |
| DHX9 | 3 | 63 |
| RRBP1 | 5 | 63 |
